# Left Atrial Myxoma Resection Through Video-Assisted Mini-Thoracotomy

**DOI:** 10.7759/cureus.114018

**Published:** 2026-08-05

**Authors:** Simon Belilty Montvelisky, Jorge Asdrubal Alfaro Chavarría, Luis Carlos Montero Salas, Bayardo Javier Robelo Pentzke, Adriana Arias González

**Affiliations:** 1 Department of Medicine, Universidad de Ciencias Médicas, San José, CRI; 2 Department of Cardiovascular Surgery, Hospital Rafael Ángel Calderón Guardia, San José, CRI; 3 Department of Pathology, Hospital Rafael Ángel Calderón Guardia, San José, CRI

**Keywords:** intracardiac myxoma, minimally invasive cardiac surgery, mini-thoracotomy, primary cardiac tumor, video-assisted thoracic surgery (vats)

## Abstract

The resection of atrial myxomas has traditionally been performed through a median sternotomy, either partial or complete, which is associated with extensive tissue disruption and larger surgical wounds. We present the case of a 71-year-old woman with a left atrial myxoma that was successfully resected using a minimally invasive approach via video-assisted right parasternal mini-thoracotomy. Direct tumor manipulation was achieved through the mini-thoracotomy, while a separate port was used for endoscopic visualization. The patient was extubated on the day of surgery and required an intensive care unit stay of five days. Continuous preoperative and postoperative telemetry monitoring demonstrated sinus rhythm throughout the entire hospital stay, with no documented arrhythmias. She was subsequently transferred to the general ward and discharged two days later without complications. These findings support that a video-assisted mini-thoracotomy represents a feasible and less invasive alternative to conventional sternotomy for selected patients.

## Introduction

Cardiac neoplasms are an uncommon occurrence. Among these, secondary cardiac tumors are far more frequent, occurring 20-30 times more often than primary cardiac neoplasms, with autopsy studies demonstrating cardiac metastases in approximately 7.1% of patients with malignancy [1]. In contrast, primary cardiac tumors are rare, with reported prevalence varying across studies depending on the population analyzed; one commonly cited estimate is approximately 0.02% in autopsy-based investigations [1]. Of all primary cardiac neoplasms, 70%-85% are benign, whereas the remaining tumors are predominantly malignant sarcomas associated with a poor prognosis [1,2]. However, this classification is based on histopathological criteria and does not necessarily reflect clinical behavior.

Historically, cardiac myxoma has been regarded as the most common primary cardiac tumor; however, recent evidence suggests that papillary fibroelastoma is now more frequently encountered, likely due to increased use and sensitivity of modern imaging techniques [3]. Cardiac myxomas are gelatinous tumors composed of an extracellular matrix with scattered spindle cells and most commonly arise from the fossa ovalis of the left atrium [3]. Macroscopically, they may present as either solid (globular) masses or papillary, friable lesions with villous projections; the latter are particularly prone to fragmentation and associated with a higher risk of systemic embolization [3]. Although histologically benign and with nonmetastatic potential, myxomas may behave in a clinically aggressive manner. Their mobility and intracardiac location can lead to obstruction of blood flow, such as occurs when a left atrial myxoma intermittently occludes the mitral valve, resulting in heart failure or sudden cardiac death; alternatively, fragmentation or surface thrombus formation may cause systemic embolization, leading to stroke, visceral infarction, or acute coronary syndrome. In addition, constitutional symptoms such as fever and weight loss are known manifestations of cardiac myxoma, which are thought to be mediated by proinflammatory cytokines secreted by the tumor, particularly interleukin-6 [3,4].

Given these potential complications, prompt surgical excision is the treatment of choice for cardiac myxomas. Traditionally, resection has been performed through a median sternotomy; however, this approach is associated with substantial surgical trauma and carries risks of complications such as wound infection, which may contribute to prolonged hospitalization and adverse clinical outcomes [5]. In response to these limitations, minimally invasive and robotic techniques have emerged as alternatives aimed at reducing surgical trauma, facilitating earlier recovery, and offering improved cosmetic outcomes. In this report, we present a hybrid minimally invasive approach combining a right mini-thoracotomy for instrument access and cardiac manipulation with mediastinal endoscopic visualization, which provided the primary visualization for complete tumor resection and septal repair.

## Case presentation

**Figure 1:**
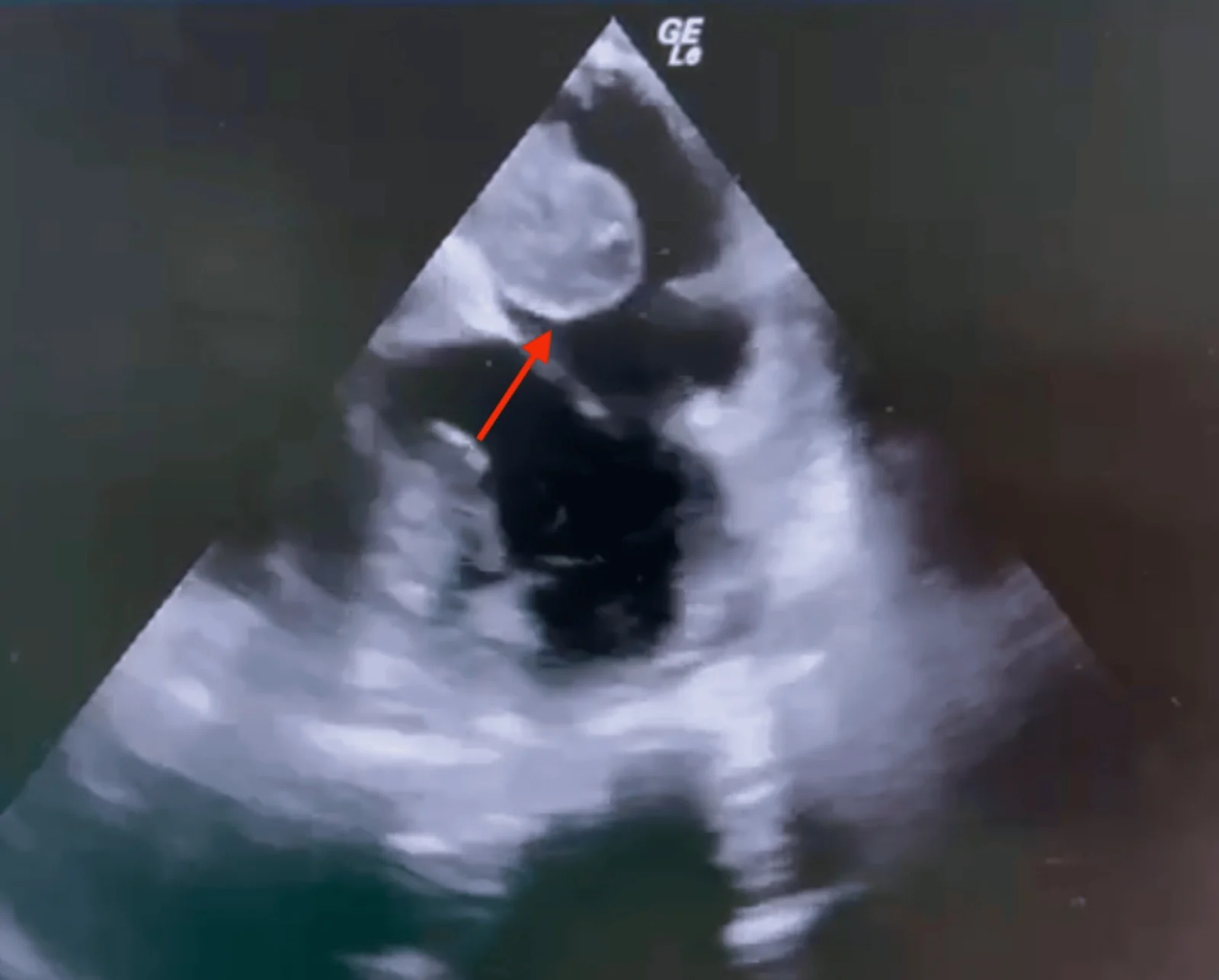
Transesophageal echocardiogram showing a well-circumscribed 3 × 2 cm echogenic left atrial mass consistent with a myxoma (arrow), attached by a narrow stalk to the interatrial septum at the fossa ovalis

A 71-year-old woman with dyslipidemia and hypertension was referred to our institution for surgical evaluation of a left atrial mass, initially identified at an outside center during the workup of palpitations and constitutional symptoms that had persisted for several months. At that time, ectopic beats were reportedly noted on clinical evaluation. Upon assessment at our center, vital signs were within normal limits, and cardiac auscultation did not reveal any abnormal rhythm or murmurs. Baseline electrocardiogram and 24-hour Holter monitoring were unremarkable, with no evidence of ectopic activity. Transthoracic echocardiography demonstrated a 3.0 × 2.0 cm heterogeneous mass in the left atrium, attached to the fossa ovalis by an avascular stalk and containing discrete calcifications. These findings were corroborated by transesophageal echocardiography, which showed a lesion suggestive of a left atrial myxoma (Figure 1).

**Figure 2:**
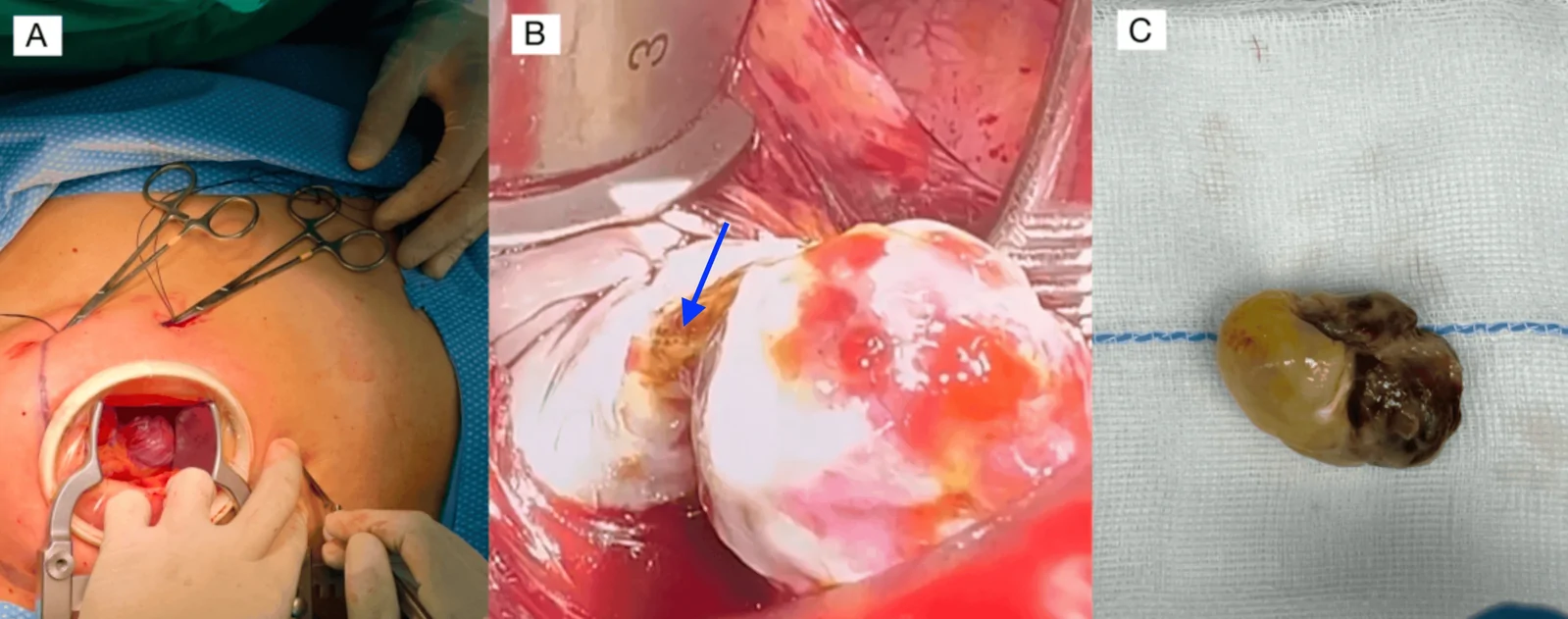
(A) Intraoperative view through a right mini-thoracotomy showing exposure of the right atrium before left atriotomy. (B) Endoscopic visualization of the left atrial cavity following atriotomy, revealing the myxoma attached to the fossa ovalis by a thin stalk (blue arrow). (C) Gross specimen excised displaying a smooth external surface and lobulated gelatinous texture typical of a cardiac myxoma

The patient was scheduled for surgical resection of the intracardiac mass using a hybrid minimally invasive approach. After induction of general anesthesia, a double-lumen endotracheal tube was inserted to allow selective right-lung collapse. The patient was positioned supine with a 30° leftward rotation to elevate the right hemithorax. A 5-cm right mini-thoracotomy was made in the fourth intercostal space just below the inframammary fold, and a retractor was inserted. A separate port was placed in the fifth intercostal space for thoracoscopic visualization and carbon dioxide insufflation, complementing the exposure provided by the mini-thoracotomy (Figure 2A). The pericardium was then opened anterior to the phrenic nerve through the mini-thoracotomy incision, exposing the ascending aorta and right atrium. Cannulation of the right femoral artery, right internal jugular vein, and right femoral vein was performed, and the patient was placed on cardiopulmonary bypass. The ascending aorta was cross-clamped for 64 minutes, and antegrade cardioplegia was administered to achieve myocardial arrest. The total cardiopulmonary bypass time was 87 minutes.

**Figure 3:**
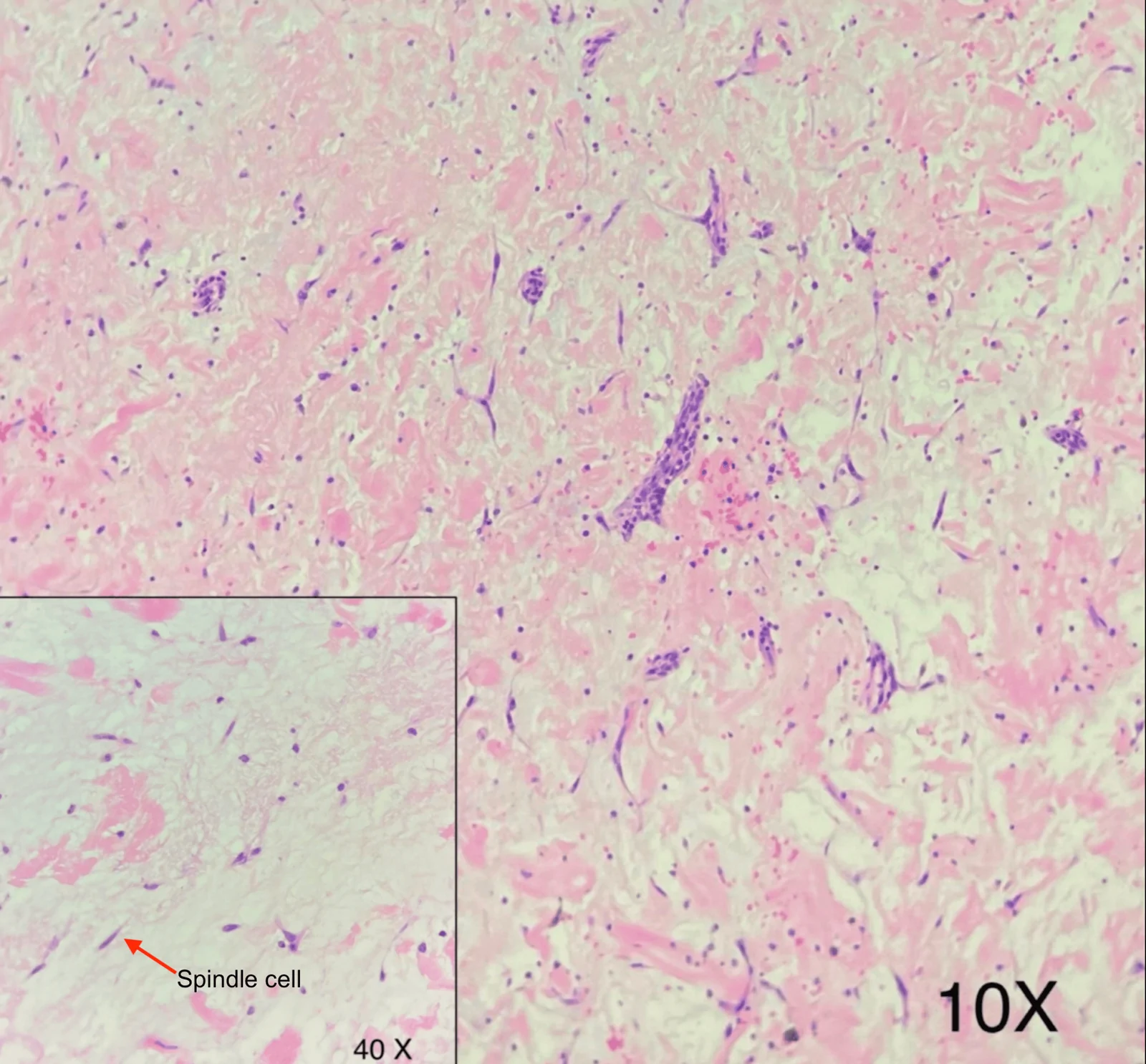
Histopathological hematoxylin and eosin section showing a myxoid stroma with sparse polygonal and stellate cells (10×). The inset (40×) highlights the characteristic spindle tumor cells

Following exposure of the right atrium, the interatrial sulcus was identified, defining the anatomical relationship between the right and left atria. A left atriotomy was then performed posteriorly along Sondergaard's line. Adequate visualization of the left atrial cavity was achieved through the combined use of the mini-thoracotomy, minimally invasive tissue retractors, and thoracoscopic assistance while preserving the surrounding structures. The tumor was identified and found to be attached to the interatrial septum by a narrow stalk at the level of the fossa ovalis (Figure 2B). Careful dissection under thoracoscopic visualization allowed the tumor to be handled through its stalk and resected en bloc with its attachment while minimizing tumor manipulation and the potential risk of fragmentation. Resection with adequate margins was performed to minimize the risk of recurrence (Figure 2C). This resulted in a small residual septal defect, which was closed by primary plication using direct sutures. Histopathological examination confirmed the diagnosis of cardiac myxoma (Figure 3).

The patient was successfully extubated on the day of surgery and experienced an uneventful recovery in the intensive care unit. A postoperative transthoracic echocardiogram demonstrated no residual intracardiac mass, no pericardial effusion, and no procedure-related abnormalities. After five postoperative days in the intensive care unit, she was transferred to the general ward and discharged home two days later in good clinical condition.

## Discussion

Surgical resection of cardiac myxomas is indicated regardless of tumor size due to the risk of thromboembolic events, intracardiac obstruction, and sudden cardiac death. Early intervention is particularly important given the unpredictable nature of embolization and the potential for acute clinical deterioration. Median sternotomy has traditionally been the standard surgical approach, offering excellent exposure and reproducibility [6]; however, it is associated with significant surgical trauma and prolonged recovery compared with less invasive techniques [7].

Over the past two decades, minimally invasive cardiac surgery has gained widespread acceptance. In appropriately selected patients, minimally invasive approaches have been associated with shorter hospital stays, reduced postoperative pain, faster recovery, and safety outcomes comparable with conventional sternotomy [8,9]. Although these data are derived primarily from valvular surgery, they illustrate the evolution and feasibility of minimally invasive cardiac surgical access. In selected patients with cardiac myxomas, these principles may similarly support a minimally invasive approach when complete tumor excision can be achieved safely.

Despite these benefits, the application of minimally invasive techniques to cardiac tumor resection remains less common. Cardiac myxomas are relatively rare, and concerns persist regarding limited operative exposure, the risk of incomplete tumor excision, and potential tumor fragmentation during manipulation. As a result, many centers continue to favor sternotomy [7]. However, published case series and reports have demonstrated that, in carefully selected patients and experienced centers, these concerns can be effectively addressed through meticulous surgical technique, with minimally invasive myxoma resection achieving adequate operative visualization, complete tumor excision, and oncologic outcomes comparable with conventional sternotomy [10].

In the present case, a hybrid approach combining a right mini-thoracotomy with thoracoscopic visualization provided adequate operative exposure, allowing careful dissection and complete en bloc tumor resection while minimizing tumor manipulation. The patient experienced no perioperative complications and an uneventful postoperative recovery, consistent with outcomes reported in the literature [10]. Importantly, this technique represents a viable minimally invasive option for appropriately selected patients and may increase the availability of minimally invasive cardiac tumor surgery in centers without robotic platforms.

However, appropriate patient selection remains critical. As no universally accepted quantitative selection criteria currently exist, candidacy for a minimally invasive approach should be determined on an individualized basis through comprehensive preoperative evaluation. Factors including tumor size, attachment site, mobility, anatomical relationships, the patient's ability to tolerate single-lung ventilation, and the experience of the surgical team should all be carefully considered.

## Conclusions

This case demonstrates the technical feasibility of a hybrid minimally invasive approach using video-assisted right mini-thoracotomy for the resection of a left atrial myxoma in a carefully selected patient. Complete en bloc tumor resection was achieved, and the patient experienced an uncomplicated perioperative course without documented postoperative arrhythmias, with the same-day extubation and an uneventful postoperative recovery. At the six-month follow-up, the patient remained asymptomatic. Although longer term surveillance remains necessary to assess recurrence risk, this case adds to the growing body of evidence supporting this approach as a viable minimally invasive option for appropriately selected patients.
